# Context dependent life-history shift in *Macrodinychus sellnicki* mites attacking a native ant host in Colombia

**DOI:** 10.1038/s41598-019-44791-2

**Published:** 2019-06-10

**Authors:** Gabriela Pérez-Lachaud, Hans Klompen, Chantal Poteaux, Carlos Santamaría, Inge Armbrecht, Guy Beugnon, Jean-Paul Lachaud

**Affiliations:** 1 Departamento de Conservación de la Biodiversidad, El Colegio de la Frontera Sur, Chetumal, 77014 Quintana Roo, Mexico; 2 Department of Evolution, Ecology and Organismal Biology, Ohio State University, Columbus, OH 43212 USA; 3 Laboratoire d’Éthologie Expérimentale et Comparée, EA 4443, Sorbonne Paris Cité, 93430 Villetaneuse, France; 4 Departamento de Biología, Grupo GEAHNA, Universidad del Valle, Cali, Colombia; 5 Centre de Recherches sur la Cognition Animale, Centre de Biologie Intégrative, Université de Toulouse UPS, CNRS-UMR5169, UPS, 31062 Toulouse, Cedex 09 France

**Keywords:** Behavioural ecology, Biodiversity, Ecological networks, Invasive species

## Abstract

Ant parasitoidism has been reported in seven of the 26 recognized species of the mite genus *Macrodinychus* (Machrodynichidae). *Macrodynichus sellnicki*, previously reported as a parasitoid of the invasive ant *Nylanderia fulva* in Colombia, is now reported, in the same region, as attacking a native host, *Ectatomma* sp. 2 (*E*. *ruidum* complex). The mite develops within the protective silk cocoon of an *Ectatomma* pupa and waits for the emergence of the young ant before leaving the cocoon, unmolested. Overall nest prevalence was relatively high (34.6% of the 52 nests containing cocoons) but pupae prevalence was low (4.0%, n = 1401 cocoons). Mite life-history (parasite or parasitoid) was context dependent, shifting according to the intensity of the attack on a same host. Contrary to the strictly parasitoidic association of *M*. *sellnicki* with *N*. *fulva*, single mite attacks against *E*. *ruidum* did not result in host killing and solitary *M*. *sellnicki* (78.6% of the cases) behaved as parasites. However, in 21.4% of the attacks (0.9% of all available host pupae) more than one mite was involved and behaved as parasitoids, draining the host of its internal fluids and killing it. This is the first association of a macrodinychid mite with a species of the subfamily Ectatomminae, and the first ant associated mite for which such a context dependent life-style shift is described.

## Introduction

Myrmecophiles (organisms living in association with ants) are able to enter ant nests and gain access to the resources therein, remaining relatively undetected by their hosts or being able to withstand or bypass host defenses^1–5^. Numerous species take advantage of resources of other organisms, and ant colonies are the hosts of an amazing diversity of organisms acting as guests, parasites or parasitoids^1,3,4,6,7^. While a large number of parasitoid species have been reported in association with ants, the precise nature of their relationships with their ant hosts often remains poorly known^8^. Mites (Acari) are ubiquitous and have colonized almost all habitats, including marine habitats^9,10^. They are probably also the most abundant and least studied group of myrmecophiles^6,11,12^. Most mite species found in the nests of ants are thought to be scavengers (feeding on fungal- and bacterial-associated debris) or parasites/predators on other myrmecophiles present in the host colony, but a few species are mutualists, protected and cared for by the ants^13^, while others are predators of ant brood or cleptoparasites, and a very small number have been confirmed as true ant parasites^11^ or parasitoids^14–19^.

Parasites and parasitoids are symbionts that negatively affect the fitness of their hosts. In this study we follow the original definition of ‘parasitoid’ given by Reuter in 1913^20^ which includes any organism where the juvenile stages parasitize a single host that is used as food source, whereas adult parasitoids are free-living. Contrary to parasites which can attack one or several individual hosts in succession but generally do not affect their survival, parasitoids attack a single host which, as a direct or indirect consequence of the parasitoid development, is killed or sterilized^7,8,21–24^. Host death as a necessity for the parasitoid to complete its development has been considered by some authors to distinguish between parasite and parasitoid^25^. However, while parasites do not require the death of their host in order to complete their development, death may occasionally occur as a consequence. Similarly, many parasitoids do not need to kill their host to complete their development if, for example, a single parasitoid attacks a large host which can afford both the parasitoid development and its own survival. This is generally the case for ant parasitoids attacking sexual brood, more specifically gyne brood^8^. In solitary parasitoids, host death may simply be a consequence of the similarity in size between host and parasitoid (parasitoid/host size ratio near to 1).

Ant parasitoidism has evolved only once in the superorder Parasitiformes, in the Uropodina (the so-called tortoise mites), within the genus *Macrodinychus* Berlese of the monotypic family Machrodinychidae (Mesostigmata)^16,18,26^. Tortoise mites are typically slow-moving inhabitants of litter and similar habitats, and are particularly diverse in social insect nests^11^ though the life-history and biology of most species is unknown. *Macrodinychus* is a pantropical genus divided in four subgenera, with 26 recognized species^19,26,27^, of which four species have been reported from the Neotropics (*M*. *mahunkai* Hirschmann, *M*. *multispinosus* Sellnick, *M*. *parallelepipedus* (Berlese), and *M*. *sellnicki* (Hirschmann & Zirngiebl-Nicol)). Up to now, the biology of only five species of *Macrodinychus* is well documented: *M*. (*Macrodinychus*) *sellnicki*^15,16^, *M*. (*Loksamacrodinychus*) *yonakuniensis* Hiramatsu^17^, *M*. (*Bregetovamacrodinychus*) *multispinosus*^18^, *M*. (*Monomacrodinychus*) *derbyensis* Brückner, Klompen & von Beeren, and *M*. (*Monomacrodinychus*) *hilpertae* Brückner, Klompen & von Beeren^19^. All of them are parasitoids of ant pupae; the immature mites parasitize a single ant pupa, eventually draining their host of all of its content and causing its death, while the adult mites are free living. This life history has made the first three species the focus of detailed studies to assess their potential as biological control agents of invasive ant species.

In a previous paper^18^, we examined published and unpublished evidence in support of the hypothesis suggested by Hirschmann, some forty years ago, that all species in the genus *Macrodinychus* might be parasitic in ant nests^28^. Hirschmann’s hypothesis was based on a number of morphological characters assumed to be associated with myrmecophily, but without any relevant data on life-history. Recently discovered life-history data support this hypothesis given that the five well documented cases noted above represent all four subgenera of *Macrodinychus*.

*Macrodinychus* mites-host associations within Formicidae are remarkably diverse. Until 2016, five species of *Macrodinychus* (*M*. *sellnicki*, *M*. *yonakuniensis*, *M*. *multispinosus*, *M*. *mahunkai*, and *M*. *shibai* Hiramatsu) had been reliably reported to be parasitoids of ant pupae belonging to three subfamilies (Dorylinae, Formicinae, and Myrmicinae)^18^. A recent report lists two new species of parasitoidic *Macrodinychus*^19^ attacking the same host, *Leptogenys distinguenda* (Emery), an Asian army ant whose larvae pupate in cocoons and which belongs to a fourth ant subfamily (Ponerinae). Moreover, two undocumented cases of mites, close to *M*. *yonakuniensis*, had been reported on the web^29^ as attacking two other ponerine ant species (*Leptogenys confucii* Forel and *Brachyponera chinensis* (Emery)). Consequently, we hypothesized that the whole genus may be comprised exclusively of ant parasitoids with low host specificity and that many more ant species could be potential hosts of parasitoid macrodinychid mites^18^. Here we both strengthen and modify this hypothesis, reporting on a new case of *M*. *sellnicki* from Colombia where the mites behave as either a parasite or as a true ant parasitoid.

Ideas on the nature of the association between *Macrodinychus* and their ant hosts have been clarified considerably since the first confirmation of pupal parasitoidism^15,16^. Early detailed reports all involved attacks on invasive ant species (*M*. *yonakuniensis* on *Pheidole megacephala* (Fabricius)^17^, *M*. *multispinosus* on *Paratrechina longicornis* (Latreille)^18^, *M*. *sellnicki* on *Nylanderia fulva* (Mayr)^15,16^). These were characterized by extremely high pupal parasitism rates (roughly 24%, 41%, and 44%, respectively, of all available pupae in the most infected populations) and, occasionally, the destruction of most of the pupae of some colonies: 90% in a *N*. *fulva* nest attacked by *M*. *sellnicki*, and 76.3% in a *Pa*. *longicornis* nest attacked by *M*. *multispinosus*. Conversely, the limited available information on attacks by these mites on native hosts indicated lower levels of prevalence of parasitoidism and we speculated that these mites may be less pathogenic on their native hosts^18^. This idea was seemingly confirmed by Brückner *et al*.^19^ who noted infection rates of less than 2% in *M*. *derbyensis* and *M*. *hilpertae* described from native ants in Malaysia. Here we describe both host association and host vulnerability characterizing the attack of *M*. *sellnicki* on what appears to be a native host, *Ectatomma* sp. 2, which belongs to the *E*. *ruidum* (Roger) complex^30^ and represents a fifth ant host subfamily (Ectatomminae) for *Macrodinychus* mites. We compare the characteristics of this association to previously described biology and behavior of the same mite on an invasive ant host, *N*. *fulva*, in the same Colombian region as in the present study (Valle del Cauca), and provide a summary of the known associations between ants and *Macrodinychus* species along with their main life-history traits.

## Results

### Ant nest composition and prevalence of parasitism

**Figure 1:**
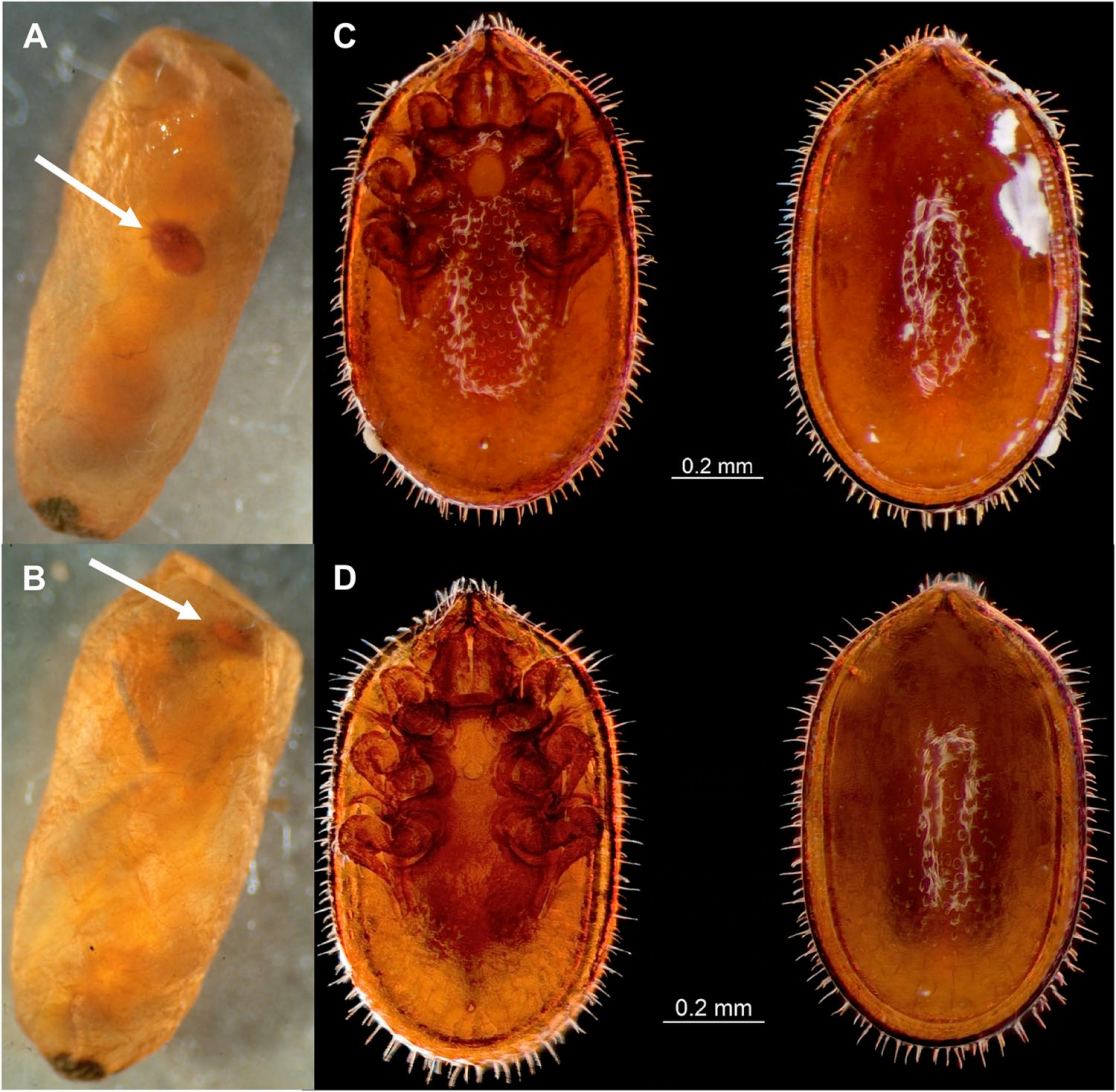
*Macrodinychus sellnicki* parasitic mite attacking an *Ectatomma* sp. 2 pupa. (**A**,**B**) General aspect of a cocoon parasitized by a mite (white arrows: an adult female individual and a deutonymph, respectively). (**C**) Ventral and dorsal views of a *M*. *sellnicki* female. (**D**) Ventral and dorsal views of a *M*. *sellnicki* male. Scale bars are 0.2 mm. Photos: (A, B) J.-P. Lachaud; (**C**,**D**) H. Bahena Basave.

**Figure 2:**
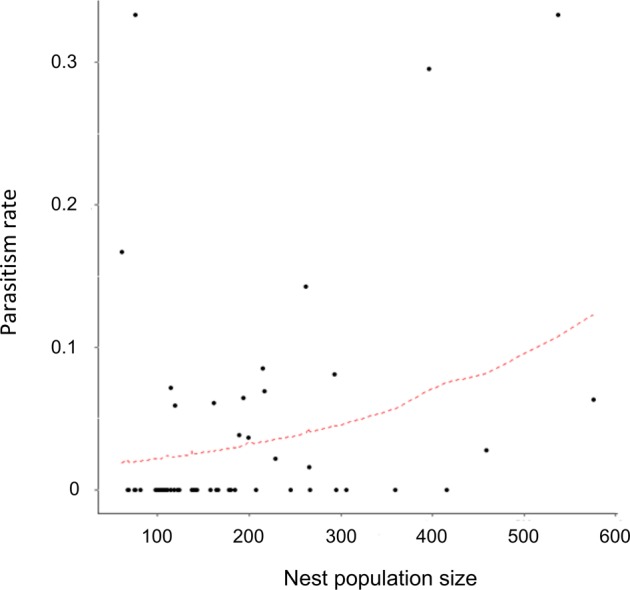
Relationship between global nest population size (brood + adults) and parasitism rate (calculated as the number of parasitized cocoons relative to the total number of available cocoons in a given nest). Dashed red line: adjusted beta binomial regression model.

**Figure 3:**
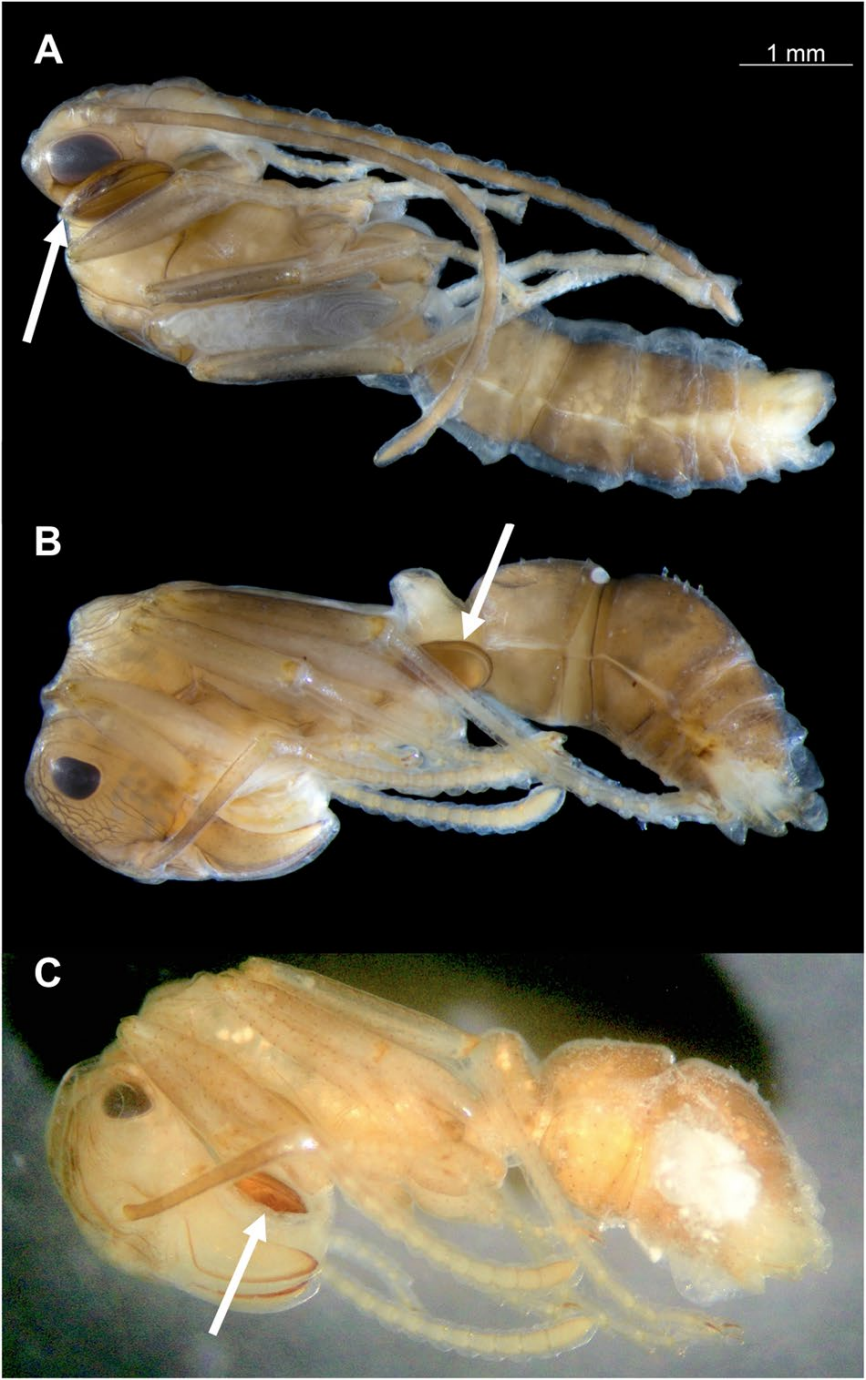
*Macrodinychus sellnicki* deutonymphs (almost adults) attached to their ant host. (**A**) An *Ectatomma* sp. 2 male. (**B**,**C**) Two *Ectatomma* sp. 2 workers. Note the different representative sites of attachment of the mites (white arrows) on their ant host: side of the head (**A**) ventral part of the gaster (**B**) or at the gular region (**C**) Scale bar is 1 mm. Photos: (**A**,**B**) H. Bahena Basave; (**C**) G. Pérez-Lachaud.

A total of 140 nests were recorded in the experimental plot, *i*.*e*. an estimated density of 3500 nests/ha, that were randomly distributed (unpubl. data). Of the 50 nests excavated in the grassland plot (see Table S1 for detailed data), only five nests contained a queen (one had two); all of the nests contained workers (mean ± SEM: 74.7 ± 4.4; range: 22–152; n = 50) and larvae (74.5 ± 11.6; range: 1–387; n = 48), most of them (90%) contained cocoons (25.5 ± 3.2; range: 2–99, n = 45), and a large proportion (66%) contained males and/or alate females (gynes). Of the eight nests collected in the forested patch (Table S1), only one was queenright but seven contained cocoons. Of the 45 nests that contained cocoons in the grassland plot, 15 (33.3%) were parasitized by *Macrodinychus* mites (Table 1, Fig. 1A–D); three of the seven nests with cocoons in the forested patch (42.9%) were also parasitized. Apart from these mites and some phoretic mites belonging to an unidentified species of *Oplitis* Berlese, no other endo- or ectoparasite (or parasitoid) of *Ectatomma* sp. 2 was found in the study sites. There was a significant positive effect of the nest population size upon the probability of a nest being parasitized (Beta binomial regression model, Z = 2.656, p < 0.001). On average, parasitized nests were more populous than unparasitized nests and differed significantly in the total nest size population (queens, gynes, males, workers, and brood pooled together; Student t test, t = 2.59, d.f. = 50, p < 0.05, Table 1) (Fig. 2). However, there was no effect of the habitat upon the probability of a nest being parasitized (forested *vs* grassland; Beta binomial regression model, Z = −1.27, n.s.) and, hence, data from both study sites were pooled. A total of 1401 cocoons were examined. Both male and worker pupae were infested (Fig. 3A–C), but not a single female pupa was found parasitized, perhaps because female pupae were very rare (only 13 female pupae *vs*. 165 male pupae and 810 worker pupae from a total of 988 cocoons for which the caste of the ant pupae could be identified). A total of 56 pupae (49 worker pupae, seven male pupae) were attacked by the mites, *i*.*e*. 4.3% and 3.0% of all the worker and male pupae, respectively (assuming that caste distribution for all the 1401 cocoons examined was the same as within the 988 cocoons for which ant caste could be identified). Overall parasitism nest prevalence was relatively high (34.6% of the nests containing cocoons, n = 52), but parasitism prevalence on ant pupae was low (only 4.0% of all available cocoons, n = 1401). A total of 81 mites were retrieved from the 56 parasitized cocoons: 38 adults (22 males, 16 females), 33 deutonymphs, 8 protonymphs, and 2 larvae. Solitary attacks were predominant and occurred in 44 of the parasitized pupae (78.6%); however, about a fifth part of all attacks (12 cases, 21.4%) were multiple: 10.7% double, 5.4% triple, and 5.4% were heavily parasitized by a total of 6 deutonymphs, 8 protonymphs and 2 larvae.

**Table 1:** Mean number (±SEM) of adults and brood items (larvae + cocoons) in unparasitized *vs*. parasitized nests of *Ectatomma* sp. 2.

	Nest condition	n	Mean number of adults	Mean brood items	Nest population size	Mean parasitized cocoons
Grassland Plot	unparasitized	30	74.7 ± 4.6	81.3 ± 9.9	156.0 ± 13.4	—
	parasitized	15	94.1 ± 9.9	136.2 ± 31.2	230.3 ± 38.2	2.7 ± 0.5
Forest Patch	unparasitized	4	98.0 ± 40.4	84.8 ± 38.8	182.8 ± 79.1	0
	parasitized	3	118.7 ± 23.4	168.0 ± 41.5	286.7 ± 57.9	5.3 ± 3.8
TOTAL	unparasitized	34	77.4 ± 6.0	81.7 ± 9.6	159.1 ± 14.4	—
	parasitized	18	98.2 ± 9.1	141.5 ± 26.6	239.7 ± 33.1	3.1 ± 0.7

Nests were collected from a 20 × 20 m grassland plot and a forested patch at the microclimatic field station of the Universidad del Valle, Cali, Colombia. Mean number of cocoons attacked by *Macrodinychus sellnicki* in parasitized nests is reported. Only nests containing cocoons were considered for this comparison.

### Natural life-history of Macrodinychus sellnicki on Ectatomma sp. 2

**Figure 4:**
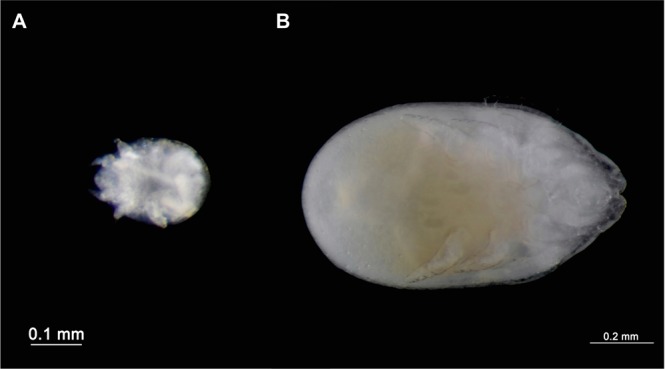
*Macrodinychus sellnicki* developmental stages. (**A**) Larva with relatively large legs allowing movement. (**B**) Fully grown deutonymph with regressed locomotor appendages. Photos: H. Bahena Basave.

All developmental stages were retrieved except the eggs, but as already reported for *M*. *sellnicki*^15,16^ and for other *Macrodinychus* species^31,32^, viviparity is strongly suspected, with active larvae rather than eggs being deposited simultaneously by females. Though actual observations of larvae searching for and attaching to an ant pupa are lacking, the mobile larva (Fig. 4A) is the presumed host searching stage^18^ and, once attached to a suitable host, the mite does not change position and waits for the host to weave its cocoon and molt. The specialized motionless feeding stages (proto- and deutonymphs) show regressed locomotor appendages (Fig. 4B), and a soft, white cuticle. Mites were found attached to various parts of the host pupa: on the side of the head or under the head capsule in the gular region, under the appendages (legs or wings), or loosely attached to the gaster (Figs 1A,B and 3A–C).

**Figure 5:**
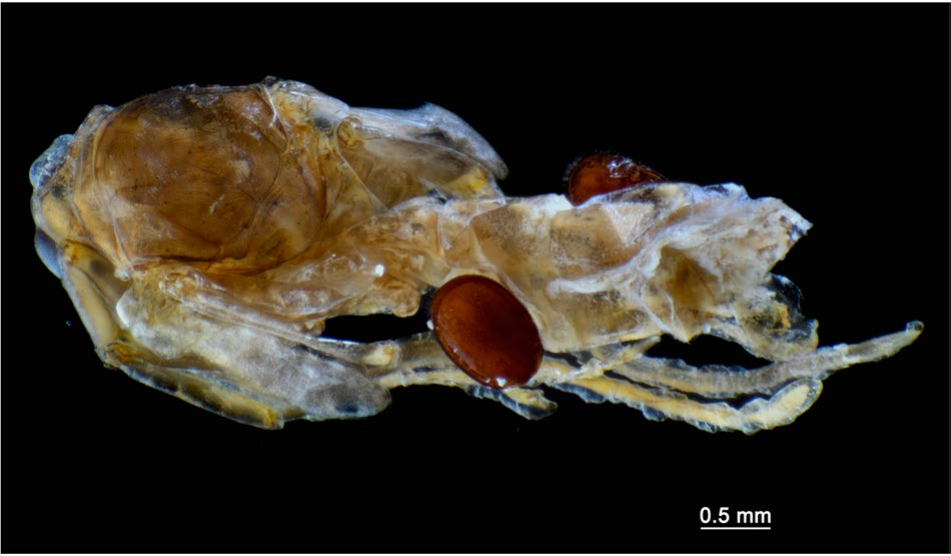
Parasitoidic behavior of *Macrodinychus sellnicki* mites. Combined attack on a worker ant pupa by six gregarious emerging adult mites (two are visible on the picture) which have almost completed their development; ant host tissues are shriveled and translucid. Photo: H. Bahena Basave.

Adult female mites are slightly larger than males (Student t test, t_body length_ = 3.37, d.f. = 11, p < 0.01; t_body width_ = 3.55, d.f. = 11, p < 0.01): 1.06 ± 0.02 mm in length and 0.63 ± 0.01 mm in width for females (n = 8) (Figs 1C and S1A) *vs*. 0.97 ± 0.01 mm in length and 0.57 ± 0.01 mm in width for males (n = 5) (Figs 1D and S1B). Pupae of *Ectatomma* sp. 2 workers measure 7.6 ± 0.1 mm in length (n = 25), and male pupae 8.0 ± 0.2 mm (n = 5). In terms of size, *Macrodinychus sellnicki* mites are quite small relatively to their *Ectatomma* hosts: mite/host size ratio is roughly 1:8 for both worker and male pupae. In most cases (78.6%) mites were found as single individuals within the ant cocoons, behaving as parasites as, apparently, they did not kill their host. Dissections of the cocoons showed that all hosts attacked by a solitary *M*. *sellnicki* were alive. Several of these parasitized pupae in an advanced stage of pigmentation were capable of some movement (for an example see Video S1) and, for some of them, the parasitic mite had already successfully completed its development and left the deutonymph exuvia upon the host. Persistence of movement was observed in most parasitized hosts which, from their general aspect, looked healthy. No other criteria were used to determine if the host was healthy. However, as previously mentioned, 21.4% of the parasitized cocoons contained two, three, or more mites (Video S2). In those cases, the general aspect of the host was dramatically different as most of their internal fluids had been sucked off by the mites, causing their death (Fig. 5). As a consequence, such gregarious mites behaved as true ectoparasitoids of the ant pupa.

## Discussion

At least a third of all known metazoan species are parasitic^33^. Parasitism (including parasitoids, macroparasites, and pathogens) has independently evolved 223 times in Animalia, with the majority of identified independent parasitic groups occurring in the Arthropoda^34^. The term parasitoid was first used by Reuter^20^ to describe a life-history intermediate between that of predators and true parasites. Contrary to parasites which do not kill their host, parasitoids eventually kill it, but unlike predators, they complete development at the expense of only one host individual. In most cases, immature stages are parasitic while adults are free-living^22^ (but see^35^). By comparison with the large bulk of potential parasites, the number of true primary parasitoids of ants is extremely limited with only approximately 750 reliable cases reported, belonging to insects (Diptera, Hymenoptera, and Strepsiptera), nematodes, and mites^6–8,24,36,37^.

Among parasitiform mites, ant parasitoids have been found only in the genus *Macrodinychus*. This new case of parasitism of *M*. *sellnicki* on *Ectatomma* sp. 2 is consistent with previous reports in terms of *Macrodinychus* development pattern and most host interactions, but differs notably in the variability of outcomes of those interactions. *Macrodinychus sellnicki* has previously been reported as a parasitoid of the invasive ant *N*. *fulva*^15,16^, invariably killing its host. Conversely, as our results show, the nature of the parasitic relationship between *M*. *sellnicki* and *Ectatomma* sp. 2, varies according to the intensity of the attack on the same host, which is highly unusual and clearly distinguishes this new association. The question of why *Macrodinychus* on *Ectatomma* may be predominantly an ectoparasite and not an ectoparasitoid is of some interest. Possible explanations might include co-evolutionary patterns limiting host vulnerability, the presence of cocoons in the ant host, and the size of the host combined with the number of mite individuals attacking the same host.

As in other very specific ant parasitoids such as encyrtid and eucharitid wasps or phorid and syrphid flies^38–41^, prevalence of parasitism by *M*. *sellnicki* upon *Ectatomma* sp. 2 was low (4.0% of cocoons), though up to 34.6% of the nests with cocoons were parasitized. When considering that only a very low fraction of the hosts died (21.4% of the parasitized pupae, *i*.*e*. only 0.9% of all available pupae), the global effect of the mite on the fitness of *Ectatomma* sp. 2 is far from severe at the population level. This might be expected for an evolutionary stable system, and is in agreement with the view of parasites of some ant societies being less virulent than those of solitary species^2^. Low prevalence of parasitism on host pupae (1.69%) was also found for the two co-occurring mite parasitoids of the ponerine army ant *L*. *distinguenda*^19^. Based on these observations, prevalence rates of *Macrodinychus* mite species on native hosts are in the same order of magnitude observed for highly specific insect parasitoids of ants.

This pattern differs radically from that previously observed for *M*. *sellnicki* on an invasive host, *N*. *fulva*, in the same geographic region. The number of *N*. *fulva* colonies infected has not been specified in the original report^15^, but the number of pupae attacked was much higher (28–44% of the pupae present in parasitized colonies). Moreover, even a single mite on the comparatively smaller and naked pupa of *N*. *fulva* will kill the host. These observations suggest co-adaptation between *Macrodinychus* mites and their native ant hosts as an explanation of their low impact. The difference between the outcome of the parasitic behavior exhibited by *M*. *sellnicki* against native and invasive host ants strengthens the hypothesis that high infestation and death rates against invasive hosts might result from an ‘invasive syndrome’ that enhances the susceptibility of these hosts to the attack of native generalist ectoparasites which are not present in their original habitat^18,42^. While this explanation seems satisfying, it is worth noting that the wasp and fly parasitoids noted above are generally very host specific while *Macrodinychus* species are not. Our results with *M*. *sellnicki* demonstrate that host specificity in this genus is not just low among species, but also at the species level. This species has now been recorded from *Ectatomma* sp. 2, *N*. *fulva*, and *Solenopsis* sp. (probably *geminata* (Fabricius)), not only three different genera, but representatives of three subfamilies of ants, casting some doubt on hypotheses of close co-adaptation in ‘natural’ systems.

A totally different explanation focuses on the presence or absence of cocoons, such as the silky cocoons of *Ectatomma* pupae. Cocoons are also formed by *L*. *distinguenda* pupae, the host of *M*. *derbyensis* and *M*. *hilpertae*. Thus, all known low-pathology associations with macrodinychid mites involve hosts that form cocoons. Cocoons have been suggested to be a successful protective envelope against fungal infections in a number of ant species^43^. Though cocoons do not prevent attacks by *M*. *sellnicki*, their presence does impose a further challenge to the parasitic mites. Adult mites do not appear to be able to cut an exit hole through the cocoon, and thus seemingly rely on the behavior of the host nurse workers to break out the cocoon and escape from the nest. Unfortunately, data on this aspect are not available for *M*. *derbyensis* and *M*. *hilpertae* because both species were essentially found in alcohol stored material some time after their collection^19^. In *M*. *sellnicki*, adult mites stay inside the cocoons and wait for the ants to emerge. Consequently, the interaction of *Macrodinychus* mites and ants which pupate in cocoons can only persist in evolutionary time if mites do not always kill the host, allowing the mites to disperse and complete their life cycle. In *Ectatomma* ants, nurse nestmates help during emergence of the newly molting adults, biting and tearing apart pieces of the cocoon, thus facilitating the exit of the young ant adult and hence that of the mite. It is unknown whether nurse ants also open cocoons when the ant occupant is dead (as in cases where *M*. *sellnicki* behaves as a parasitoid), or whether they just throw these cocoons away as happens in *E*. *ruidum* and *E*. *tuberculatum* (Olivier) with cocoons parasitized by eucharitid wasps^44,45^. In the case of eucharitids, however, the emerging wasps can cut an exit hole on their own, taking advantage of the prophylactic behavior of the ants to exit the nest unharmed^45^.

If the presence of cocoons influences prevalence of the mites, that might partially explain why *M*. *sellnicki* parasitism is far more aggressive on myrmicine and formicine ants whose pupae are naked. In Colombia, up to 90% of the naked pupae of a single colony of the invasive *N*. *fulva* may be killed by this mite, with an average of 44% in the most infested population^15^. Moreover, even when attacking the naked pupae of the native host *Solenopsis* sp. (probably *geminata*), the prevalence of pupae parasitism was much higher than for *Ectatomma* sp. 2, with values reaching up to 30% in the most infested locality (see Table 2). Similarly, in Okinawa Island, 92% of the nests of *Ph*. *megacephala* were attacked by *M*. *yonakuniensis* with a general prevalence of parasitism on naked pupae of 15.5% but a prevalence of parasitism on major worker pupae of up to 93.6% in one population^17^. In Mexico, *M*. *multispinosus* also exacted high fitness costs from *Pa*. *longicornis* colonies by killing on average 26.2% of the global population of available naked pupae, and up to 41.3% in the most infested population^18^ (Table 2).

**Table 2:** Known parasitic associations of the mite genus *Macrodinychus* with ants. For prevalence, values can refer either to the proportion of nests attacked (N) or to the proportion of pupae attacked (P). Development of pupae as naked pupae or within a cocoon is noted (C−) and (C+), respectively.

Mite species	Host species	Host subfamily	Life-style	Mite/host size ratio	Prevalence (%)	Host status	Location	References
*M*. *derbyensis*	*Leptogenys distinguenda*	Ponerinae	ectoparasitoid	≈1:3	≤1.7 (P)	native (C+)	Malaysia	^19^
*M*. *hilpertae*	*Leptogenys distinguenda*	Ponerinae	ectoparasitoid	1:3–1:4	≤1.7 (P)	native (C+)	Malaysia	^19^
*M*. *mahunkai*	*Labidus coecus*	Dorylinae	ectoparasitoid	?	?	native (C+)	Ecuador	^18^
*M*. *multispinosus*	*Paratrechina longicornis*	Formicinae	ectoparasitoid	1:2–1:3	47.1 (N) − 26.2 (P)	invasive (C−)	Mexico	^18^
	*Nylanderia fulva*	Formicinae	ectoparasitoid	≈1:4	?	invasive (C−)	Colombia	^18^
*M*. *sellnicki*	*Nylanderia fulva*	Formicinae	ectoparasitoid	1:3–1:4	28–44 (P)	invasive (C−)	Colombia	^15^
	*Solenopsis* (*geminata*?)	Myrmicinae	ectoparasitoid	?	2.5–30 (N)	native (C−)	Colombia	^15^
	*Ectatomma* sp. 2	Ectatomminae	ectoparasite/ectoparasitoid	≈1:8	34.6 (N) − 4.0 (P)	native (C+)	Colombia	this study
*M*. *shibai*	? (host lost)	?	ectoparasitoid	?	?	?	Philippines	^18^
*M*. *yonakuniensis*	*Pheidole megacephala*	Myrmicinae	ectoparasitoid	≈1:3–1:4	92 (N) − 15.5 (P)	invasive (C−)	Japan, New Caledonia	^17^ Le Breton pers. com.
	*Pheidole noda*	Myrmicinae	ectoparasitoid	?	?	native (C−)	Japan	^17^
*M*. sp. 1 ca. *yonakuniensis*	*Leptogenys confucii*	Ponerinae	ectoparasitoid	?	?	native (C+)	Japan	^29^
*M*. sp. 2 ca. *yonakuniensis*	*Brachyponera chinensis*	Ponerinae	ectoparasitoid	?	?	native (C+)	Japan	^29^

Cocoons may also affect attachment site specificity. While *M*. *sellnicki* attacking *N*. *fulva* attached almost exclusively to the gular region^15^ and *M*. *yonakuniensis* and *M*. *multispinosus* almost exclusively to the ventral surface of the gaster of their respective host^17,18^, *M*. *sellnicki* attachment on *Ectatomma* sp. 2 is not very specific and can occur at multiple sites on the host body, though most of the time mites were found at the gular region. The presence of a protective cocoon, preventing the developing mite from falling off the pupa, may have voided the need for an optimal attachment site. Notably, presence of *Macrodinychus* mites on a pupa always seems to cause deformation in the host body, forming a protective cavity.

Finally, the different outcomes of associations of mites and ants may be a simple artifact of size, specifically the size of host pupae relative to the mites. Most hosts attacked by *Macrodinychus* species are relatively small (mite/host size ratio varying roughly between 1:2 and 1:4) (Table 2). By contrast, *Ectatomma* sp. 2 ants are quite large, changing the mite/ant size ratio to about 1:8. As a result, the attack by a single specimen of *M*. *sellnicki* may not be sufficiently draining to affect host survivorship, leaving solitary mites essentially behaving as ectoparasites. However, when multiple individuals attack the same host, the intensity of their combined action does lead to the host death by draining all of the host content. It is worth noting that attacks by multiple mites on a single host are rare in *Macrodinychus*. In naked pupae, attachment to non-specific sites may lead to detachment when pupae are cleaned or transported by nurse workers. Multiple attacks have been reported for M. *sellnicki* attacking *N*. *fulva* male pupae^15^ and a unique case of double infestation by deutonymphs has been reported for *M*. *multispinosus* on *Pa*. *longicornis*^18^. Thus, mite/host size ratio, presence/absence of cocoons, and perhaps co-evolutionary patterns (e.g. more aggressive mite removal by native hosts, as documented for *L*. *distinguenda*^19^) may all contribute to the observed context dependent effects of *M*. *sellnicki* associations with *Ectatomma* sp. 2.

Until now, the *E*. *ruidum* complex has only been reported as host of parasitoids belonging to eucharitid wasps, phorid flies and a species of nematode^24^. This is the first documented report of uropodine ectoparasitoid mites associated with Ectatomminae, the fifth ant subfamily associated with parasitoidic macrodinychids. Such an occurrence in *Ectatomma* sp. 2, which has been the focus of numerous previous studies in different Neotropical zones, might be related to some undetermined ecological factors or to the particular social structure exhibited by this species in the study site. Nest relocation is infrequent in this species, but more common in the study zone (CS, unpubl. data). Furthermore, polydomy is strongly suspected, and transport of larvae and cocoons between nests has been regularly observed in the study plot (unpubl. data). The risk-spreading benefit of polydomy in the studied population could have the potential of isolating parasites^46^. These behavioral traits may favor spread of the mites among nests. However, as with many other associations involving very tiny organisms, especially those hidden within a cocoon^41,47^, it is also possible that these symbiotic associations had been quite simply overlooked. More data are needed about the specificity of the *Macrodinychus*/*Ectatomma* association, the temporal distribution of mites in *Ectatomma* nests, and the behavioral interactions with their host ants (if any).

## Material and Methods

### Natural life-history of the host

*Ectatomma* Smith (Formicidae: Ectatomminae) is one of the most frequently collected genera of Neotropical ants, with 15 species currently recognized^48,49^. The genus includes relatively large ants (up to 15 mm in length), all endemic to the neotropical region^50^. Historically, the dominant *E*. *ruidum* had been considered as a single, though very variable, species distributed from central Mexico to northern Brazil, from sea level to 2200 m asl^51^. However, molecular analyses have recently shown that the species is a complex of at least three cryptic species with very little morphological variation^30,49,52,53^. Two of these species have a wide distribution in the neotropics; the first one corresponds to the syntype of *E*. *ruidum* described by Roger^54^ and the second, *Ectatomma* sp. 2, is considered as a new, still undescribed species. The third species, *Ectatomma* sp. 3, also undescribed, is endemic of the southern Mexican Pacific coast of Oaxaca. Based on confirmed records, *Ectatomma* sp. 2 extends from Tamaulipas and Nayarit on the Atlantic and Pacific coasts of Mexico, to southwestern Ecuador^30^. This species nests in the soil, and colonies show highly variable social structure and behavior^49^.

Molecular analysis of the ant species concerned in the present study was performed within the framework of a previous work focused on delimiting the species boundaries of the *E*. *ruidum* complex^30^. Barcoding confirmed that the population sampled in the present study corresponded to *Ectatomma* sp. 2 (Genbank accession number: KU570627.1)^30^. However, microgynes, generally present in the Mexican populations (referred to as *E*. *ruidum*^55,56^) are lacking in the Colombian population studied here, which is also characterized by an atypical aleatory nest distribution and a likely polydomous nest structure^57,58^. The position of a nest both in the environment and in the network of dynamic nests in the case of a polydomous species is thought to affect access to resources and ultimately the fitness of the individuals within the nest^59,60^. Workers from the same colony, but inhabiting different nests, therefore have different access to resources and may also have different probabilities of being parasitized^46^. Due to the putative polydomic structure of the population studied, we preferred to evaluate parasitism at the nest level rather than at the colony level, and the term ‘nest’ has been preferred to ‘colony’ throughout the text.

### Sampling

*Macrodinychus* mites were found during a large-scale field study concerning thievery and potential polydomy in *Ectatomma* sp. 2, conducted on 6–16 June 2016 at the microclimatic field station of the Universidad del Valle (Campus Meléndez, Santiago de Cali) in Colombia (3°22′25.42″N, 76°31′50.68″W, 970 m asl). Mean annual temperature is 24.1 °C and average relative humidity 73%; average annual rainfall is around 1500 mm with two rainfall peaks, from March to May and from September to November^61^.

A 20 × 20 m plot was delimited in a grassland which was continuously mowed down, and all *Ectatomma* sp. 2 nests located in the plot were censused. Of these, 50 nests were excavated and both adults and immatures (eggs, larvae and cocoons) were individually inspected under a stereomicroscope for any exterior sign of parasitism. Cocoons from each nest were then isolated in vials tapered with a cotton plug and later dissected (Table S1). All of the material was carefully revised for initial stages of development of the mites attached to the ant pupae; their position and number were recorded. Eight supplementary nests were excavated in a small patch of tropical dry forest, at 300 m apart from the grassland plot, and the material was examined as above.

### Parasitism prevalence and mite/host size ratio

Actual percentages of mite parasitism were calculated for each nest as the number of parasitized cocoons relative to the total number of available cocoons (Table S1). To explore for an effect of nest population size and site (grassland plot *vs* forested patch) upon the probability of a given nest being parasitized, and because of overdispersion of our data, a beta binomial regression model was adjusted^62^. The beta binomial model incorporates the fact that data are the product of Bernoulli trials with unequal probability of events. Since the parameters of the model are estimated from a sample, the Wald test can be used to test the true value of the parameter based on the sample estimate. The Z statistic reported here is a Wald type statistic, that corresponds to an asymptotic approach to the normal distribution. Under the Wald statistical test, the maximum likelihood estimate of the parameter of interest is compared with the proposed value with the assumption that the difference between the two will be approximately normally distributed. Statistics were performed using R^63^.

Mites and their hosts were measured under a stereomicroscope with a micrometer. Mite body length along the dorsum, not including the gnathosoma, and maximum body width were measured. Total length of ant pupae in lateral profile was measured as an estimation of host size and used to calculate adult parasitoid/host size ratios, considering the average length of adult mites of both sexes.

Mite specimens were cleared in lactic acid, dissected, and mounted in Hoyer’s medium on microscope slides for identification. In addition, two specimens were mounted on a stub, critical point dried, sputtered with gold, and observed with a Jeol JSM-6010PLUS/LA scanning electron microscope. Our specimens showed identical peritrematal, dorsal shield, and setal structure and setal distribution patterns (major species-level characters in *Macrodinychus*^27^) as specimens of *M*. *sellnicki* from *N*. *fulva* (OSAL 0046552 (♀), 0046549, 0046517 (♂)), strongly suggesting that they too belong to *M*. *sellnicki*. Voucher specimens of both mites and ants were deposited in the collection of the Museo de Entomología de la Universidad del Valle (MUSENUV) in Colombia, the formicid collection of El Colegio de la Frontera Sur in Chetumal (Mexico) (ECO-CH-F: F-2198–2201), and at the Acarology Laboratory of Ohio State University in Columbus (USA) (OSAL 0117964 (♀), 0117965 (♂); https://acarology.osu.edu/database). Collection and transport of arthropod specimens were authorized by the Autoridad Nacional de Licencias Ambientales (Permit No. 1070 to the Universidad del Valle). The collection did not involve endangered or protected species. Research and field work complied with the current laws of Colombia.

## Supplementary information

Supplementary Information

Single attack by a Macrodinychus sellnicki mite

Multiple attack by Macrodinychus sellnicki mites
