# Supplementary material for: Context dependent life-history shift in Macrodinychus sellnicki mites attacking a native ant host in Colombia
Source: Sci Rep. 2019 Jun 10;9:8394. doi: 10.1038/s41598-019-44791-2 (PMC6557818; doi:10.1038/s41598-019-44791-2)
Supplement: Supplementary file 1 — Supplementary Information [file 41598_2019_44791_MOESM1_ESM.pdf]

Supplementary Information

Context dependent life-history shift in *Macrodinychus sellnicki* mites attacking a native ant host in Colombia

Gabriela Pérez-Lachaud[1], Hans Klompen[2], Chantal Poteaux[3], Carlos Santamaría[4], Inge Armbrecht[4], Guy Beugnon[5], Jean-Paul Lachaud[1,5,*]

[1] Departamento de Conservación de la Biodiversidad, El Colegio de la Frontera Sur, Chetumal 77014, Quintana Roo, Mexico

[2] Department of Evolution, Ecology and Organismal Biology, Ohio State University, Columbus OH 43212, USA

[3] Laboratoire d'Éthologie Expérimentale et Comparée, EA 4443, Sorbonne Paris Cité, 93430 Villetaneuse, France

[4] Departamento de Biología, Grupo GEAHNA, Universidad del Valle, Cali, Colombia

[5] Centre de Recherches sur la Cognition Animale, Centre de Biologie Intégrative, Université de Toulouse UPS, CNRS-UMR5169, UPS, 31062 Toulouse Cedex 09, France

Content

Figure S1. Scanning electron micrographs of *Macrodinychus sellnicki* adults.

Table S1. Content of the *Ectatomma* sp. 2 nests excavated and results of cocoon dissections.

Video S1. An *Ectatomma* sp. 2 male pupa remains alive after successful parasitism by a single *Macrodinychus sellnicki* mite.

Video S2. Multiple attack by *Macrodinychus sellnicki* on a worker host pupa.

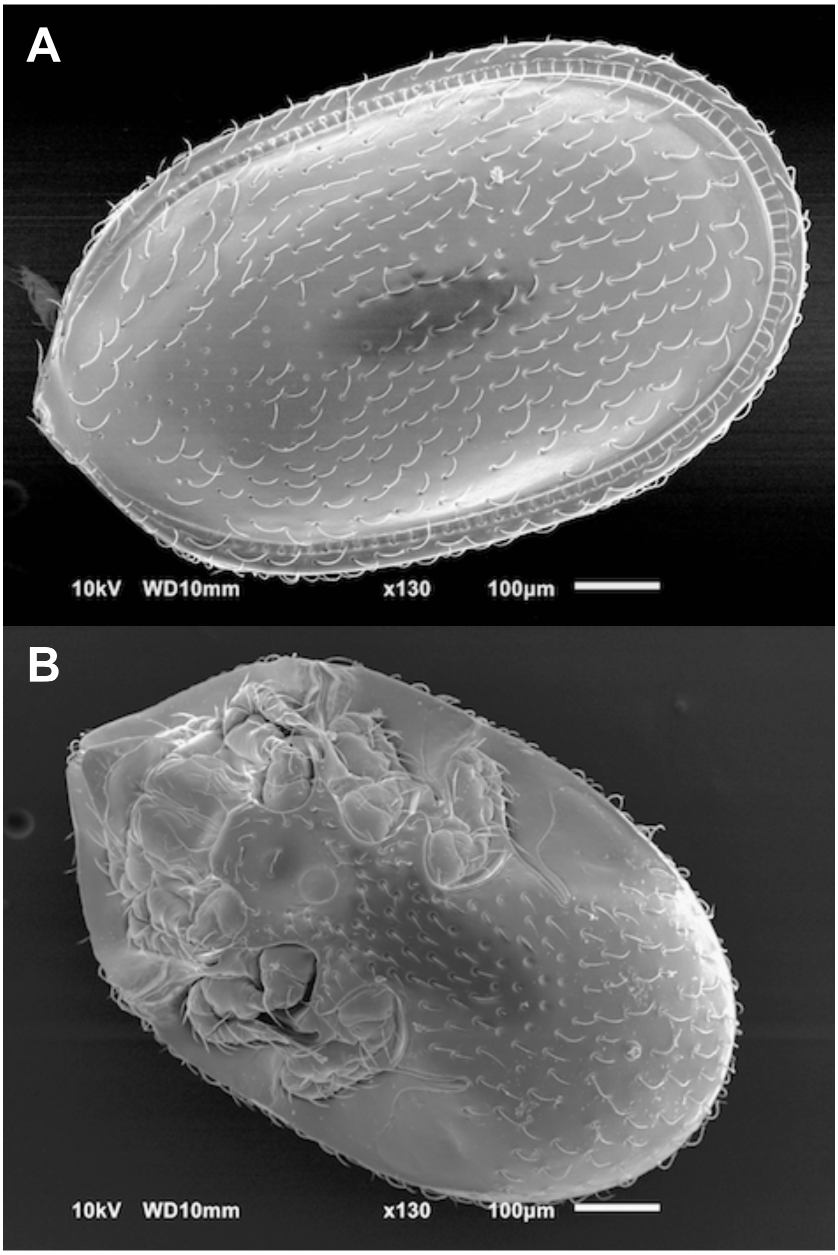

Figure S1. Scanning electron micrographs of *Macrodinychus sellnicki* adults. (A) Dorsal view of a female. (B) Ventral view of a male. Note that the legs may be withdrawn into special depressions (pedofossae) enhancing adult mite protection against its host. Scale bars are 100 µm. Photos: G. Pérez-Lachaud & M. Elias-Gutiérrez.

Table S1. Content of the *Ectatomma* sp. 2 nests excavated and results of cocoon dissections. Nests were excavated and collected at the Biological Station (Universidad del Valle, Cali, Colombia, 11-16 June, 2016). Cocoons were dissected under a stereomicroscope.

Sampling									Dissections						
	Nest #	queens	gynes	males	workers	cocoons	larvae	eggs	Crushed pupae	pre-pupae	male pupae	female pupae	worker pupae	parasitized cooons	parasitism rate (%)
Grassland	6	0	0	2	45	14	15	0	0	7	0	0	7	0	0.00
Plot	9	2	0	0	124	63	387	> 60	0	13	9	0	37	4	6.35
	11	0	1	7	75	52	45	0	0	22	5	0	25	0	0.00
	11b	0	0	6	102	47	60	0	0	13	5	1	24	4	8.51
	12b	0	1	2	57	-	2	0	-	-	-	-	-	-	-
	13	0	0	3	107	58	49	0	0	13	2	2	37	4	6.90
	14	0	0	0	64	20	34	0	1	11	0	0	8	0	0.00
	15	0	2	3	66	25	5	0	0	2	0	0	23	0	0.00
	16	0	1	0	86	62	45	0	1	19	2	2	34	4	6.45
	17	0	4	5	111	99	74	0	4	25	19	0	43	8	8.08
	19	0	10	5	102	46	66	0	0	15	6	2	22	1	2.17
	20	0	6	4	57	17	35	0	0	5	0	1	10	1	5.88
	32	0	0	9	87	34	28	0	0	9	10	0	15	0	0;00
	48	0	3	5	66	18	50	0	0	5	4	2	7	0	0.00
	49	0	1	2	42	6	17	0	0	1	2	1	2	0	0.00
	51	0	2	5	95	17	24	0	0	4	5	0	8	0	0.00
	52	0	1	7	78	14	64	0	0	6	3	0	5	0	0.00
	53	0	0	0	56	14	44	0	2	5	3	0	3	1	7.14
	54	0	1	0	86	8	150	6	0	3	5	0	0	0	0.00
	55	0	0	20	100	14	128	0	0	2	4	0	6	2	14.29
	61	0	0	11	22	6	70	1	0	2	2	0	2	0	0.00
	62	0	0	0	140	30	136	0	0	13	5	0	12	0	0.00
	63	0	0	7	78	30	51	0	5	8	6	0	11	0	0.00
	64	0	0	9	46	23	36	0	6	6	1	0	10	0	0.00
	69	0	0	0	104	-	104	0	-	-	-	-	-	-	-
	70	0	0	0	35	-	28	0	-	-	-	-	-	-	-

	71	0	0	0	40	3	38	0	0	1	0	0	2	0	0.00
	72	1	0	0	118	25	215	12	0	5	0	0	20	0	0.00
	73	0	0	0	80	25	190	7	1	12	0	0	12	0	0.00
	74	0	0	1	65	14	42	30	0	2	1	0	11	0	0.00
	75	0	2	0	47	-	1	0	-	-	-	-	-	-	-
	76	0	0	5	50	33	73	0	0	16	3	0	12	2	6.06
	78	0	4	4	85	26	70	0	0	12	4	0	9	1	3.85
	79	0	0	2	53	10	38	0	1	4	2	0	3	0	0.00
	90	0	0	3	68	19	34	0	0	3	7	0	9	0	0.00
	92	1	0	0	136	72	> 250	0	0	10	8	0	52	2	2.78
	95	0	4	0	86	59	58	0	0	10	5	0	44	0	0.00
	103	0	0	1	72	4	60	0	0	0	0	0	4	0	0.00
	114	0	1	1	91	22	63	0	0	8	0	0	14	0	0.00
	115	0	0	0	77	33	74	0	0	8	4	0	21	0	0.00
	116	0	0	0	47	2	26	0	0	0	1	0	1	0	0.00
	117	0	0	0	27	-	5	4	-	-	-	-	-	-	-
	118	1	0	0	125	2	138	7	0	2	0	0	0	0	0.00
	119	0	0	1	63	7	69	> 10	0	2	1	0	4	0	0.00
	120	0	0	0	48	27	31	0	1	7	2	0	17	0	0.00
	121	0	0	0	35	6	35	0	0	0	1	0	3	2	33.33
	126	0	0	2	24	6	29	0	0	2	2	0	1	1	16.67
	127	0	0	0	56	15	27	0	0	4	1	0	10	0	0.00
	128	1	0	1	152	9	374	2	0	4	1	0	1	3	33.33
	129	0	1	6	60	10	37	0	0	5	3	0	2	0	0.00
Forested Patch	A	1	0	6	210	48	151	> 40	0	11	9	0	28	0	0.00
	B	0	0	2	69	19	47	0	0	6	1	0	12	0	0.00
	C	0	0	1	37	-	29	2	-	-	-	-	-	-	-
	D	0	0	2	35	19	11	0	0	8	0	0	11	0	0.00
	E	0	0	0	72	55	72	0	0	15	0	0	38	2	3.64
	MSt1	0	0	23	116	62	64	?	0	13	2	0	46	1	1.61
	MSt2	0	0	17	128	44	207	?	0	9	2	2	18	13	29.55
	MSt3	0	6	6	55	8	36	?	0	3	0	0	5	0	0.00

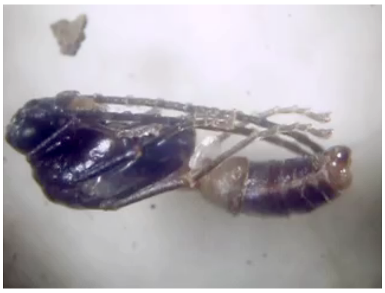

Video S1. An *Ectatomma* sp. 2 male pupa remains alive after successful parasitism by a single *Macrodinychus sellnicki* mite. Note that the host is capable of some movement. The whitish structure on the ant leg is the deutonymph mite exuvia left by the adult mite after molting.

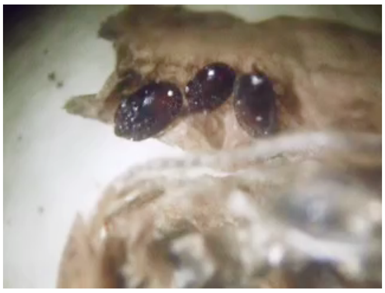

Video S2. Multiple attack by *Macrodinychus sellnicki* on a worker host pupa. Three mites which have completed their adult molt are visible on the remains of a dissected cocoon, one is trying to escape.
